# Implementing trachoma control programmes in marginalised populations in Tanzania: A qualitative study exploring the experiences and perspectives of key stakeholders

**DOI:** 10.1371/journal.pntd.0009727

**Published:** 2021-09-10

**Authors:** Kaki Tsang, Gilles de Wildt, Upendo Mwingira, Tara B. Mtuy

**Affiliations:** 1 The Department of Population Sciences and Humanities, College of Medical and Dental Sciences, University of Birmingham, Birmingham, United Kingdom; 2 RTI International, Washington DC, United States of America; 3 NTD Control Programme, National Institute for Medical Research, Dar es Salaam, Tanzania; 4 Department of Global Health and Development, London School of Hygiene & Tropical Medicine, London, United Kingdom; London School of Hygiene&Tropical Medicine, UNITED KINGDOM

## Abstract

**Background:**

Despite aspects of the SAFE strategy for reducing trachoma in Tanzania have been somewhat successful, the disease still persists in marginalised communities even with repeated trachoma control interventions. This study aims to understand the facilitators and barriers associated with implementing trachoma control programmes in these communities, from the perspective of non-governmental organisations (NGOs).

**Methods:**

Participants were the representatives of NGOs who had knowledge and experience in the implementation of trachoma control programmes. Data was collected using in-depth, semi-structured interviews guided by a topic guide, which was updated after each interview using a constant comparative method. Interviews were audio-recorded and then transcribed verbatim. Thematic analysis was done inductively. Codes were generated from the transcripts and then clustered into themes.

**Findings:**

The context within marginalised communities often acted as a perceived barrier to successful implementation of control programmes. This included poor environmental cleanliness, lack of trust, poor disease knowledge and traditional lifestyles. Community values could either be a facilitator or a barrier, depending on the scenario. The anatomical location of the disease and the poor understanding of the disease progression also served as barriers. Considerations affecting decision-making among NGO’s include financial feasibility, community needs and whether the quality of the intervention could be improved. NGOs felt that the collaboration and the opportunity to learn from other organisations were beneficial aspects of having different actors. However, this also resulted in variability in the effectiveness of interventions between districts.

**Conclusion:**

NGOs should focus on behaviour change and health education that is tailored to marginalised communities and seek innovative ways to implement trachoma intervention programmes whilst being minimally intrusive to the traditional way of life. Partners should also implement ways to ensure high quality programmes are being provided, by increasing staff accountability and compensating volunteers fairly.

## Author summary

Trachoma is a neglected tropical disease caused by the bacterium *Chlamydia trachomatis* and can result in blindness if left untreated. The World Health Organisation devised the SAFE strategy (Surgery, Antibiotics, Facial Cleanliness and Environmental Improvements) to combat this disease. In Tanzania, many trachoma control interventions are implemented by non-governmental organisations (NGOs). The disease still persists in areas with marginalised, including Maasai, despite repeated interventions. These communities are often difficult to reach due to their livelihoods and local beliefs. This study investigates the facilitators and barriers to implementing interventions within hard to reach communities from the perspectives of NGOs. Findings will provide insight on how NGOs implement and ensure their programmes are effective, whilst being mindful of the intrinsic factors important to the community, which will inform improvements and alterations in trachoma interventions.

## Introduction

Trachoma is an ocular infection caused by *Chlamydia trachomatis* and is a neglected tropical disease found predominantly in developing countries [1]. As of 2020, the World Health Organisation (WHO) estimates that 137 million people worldwide are infected with trachoma, making it the leading cause of infectious blindness globally and a public health priority [2]. The SAFE strategy, which stands for Surgery (S), Antibiotics (A), Facial cleanliness (F) and Environmental change (E), was devised with the goal of eliminating trachoma by 2020[3]. Despite this, the disease is currently still endemic in 44 countries [2].

Surgery has been repeatedly demonstrated to be beneficial in preventing the worsening of trichiasis and preserves visual acuity, as well as reducing pain and associated symptoms [4,5]. Antibiotics in the form of mass drug administration (MDA), a common method of drug delivery for neglected tropical diseases, is effective in reducing the prevalence of active trachoma and therefore the transmission of the disease [6–9]. The F and E components of the strategy focus more on the primary prevention of the disease and relies on broader community hygiene interventions and behavioural change [10]. There is evidence for the benefit of improving water supply and sanitation in reducing the risk of trachoma [11]. A few studies have found that having health education on improving hygiene practices are correlated with a lower trachoma prevalence [12,13]. In various countries where the full SAFE strategy has been implemented effectively, trachoma has often successfully been eliminated, with the most recent success being in Ghana as of 2019[14]. However, one of the caveats to the effectiveness of the SAFE strategy is that all four components need to be implemented simultaneously in order to successfully eliminate trachoma.

Aspects of the SAFE strategy have been successful in some districts in Tanzania [15]. However, it has been recognised that some districts with marginalised communities, mostly consisting of the Maasai populations, have not seen the same success [16]. In some of these predominantly Maasai districts, the prevalence of active infection, including the presence of follicles and papillary inflammation, has been found to be 33.6% and 31.6% respectively in children aged 6 to 10 years old [17], which is far greater than the WHO elimination threshold of <5% [2]. The Maasai ethnic group occupies the northern regions of Tanzania and the southern regions of Kenya [18]. Many of the Maasai people still live by their traditional pastoralist lifestyles, rearing and living in close proximity to their cattle, as well as following a semi-nomadic way of life.

Trachoma control programmes in Tanzania are implemented by the Tanzania National Neglected Tropical Diseases (NTD) Control Programme in collaboration with non-governmental organisations (NGOs). The organisations might only support one of the components of SAFE, meaning that the Tanzania National NTD Control Programme coordinates activities to ensure endemic districts are covered with applicable components of SAFE [19].

A MEDLINE literature review has shown that there is a gap in the literature, as there are few previous studies investigating the perspectives of NGOs on the implementation of trachoma interventions. One study looked at the barriers to implementation of SAFE in Australia in Aboriginal communities from the perspective of healthcare staff, however, owing to the completely different context and environment, it is difficult to say whether the findings of the study are generalisable to the Tanzania context [20]. Therefore, this study aims to understand programme implementation by NGOs on a broader level in these communities in Tanzania. The study explores the facilitators and barriers of implementing trachoma interventions in the context of marginalised communities in Tanzania, which are predominantly inhabited by the Maasai, the effect of having multiple actors involved in delivering trachoma interventions and the factors that influence decision-making relevant to the programme.

## Methods

### Ethics statement

Favourable opinions were obtained from three ethical committees: The University of Birmingham Internal Research Ethics Committee, the London School of Hygiene and Tropical Medicine Research Ethics Committee, and the National Institute of Medical Research in Tanzania. Data was stored in accordance with both the European GDPR data protection policy and the policies of the universities from which ethical approvals were obtained. Informed written and verbal consent was obtained from all participants. Permission for the data to be audio-recorded was also obtained from each participant.

#### Setting

A qualitative study was deemed most appropriate to investigate the experiences and perspectives of the key stakeholders implementing trachoma interventions. Semi-structured interviews with the representatives of the organisations involved with trachoma interventions in marginalised communities, primarily comprising of Maasai populations, were conducted during February and March 2019.

The research team was based in Moshi, Tanzania. For the stakeholders with offices in Tanzania, interviews were conducted in person. Interviews with stakeholders with foreign offices were conducted via a Skype call. On a regular basis, the National NTD Control Programme assigns districts to the stakeholders to ensure that trachoma endemic areas are covered with the appropriate control programme.

#### Sampling and recruitment

Purposive sampling was the primary method used to recruit study participants, owing to the limited number of stakeholders and therefore potential participants. Relevant organisations were identified from a list of the members of the International Coalition for Trachoma Control to see whether they were implementing trachoma interventions in Tanzania. Snowball sampling was used to identify additional suitable and willing participants. Participants were recruited based on their knowledge and experience in the implementation of trachoma interventions in Maasai or marginalised communities in Tanzania and whether they had a working level of English. Participants were excluded if they would not or could not give informed consent. Recruitment ended when all the willing stakeholders that fit the sampling criteria were interviewed.

Participants were contacted and given information on the study via email and were followed up a few days later if no reply was received. All the participants were literate and gave informed written consent prior to the interview. The time and location of the interviews were chosen to suit the participants. All of the interviews, either in-person and Skype, took place in a private and quiet room and were conducted by the lead author.

#### Data collection

A flexible interview guide was used during all the interviews and developed to improve understanding on the four study objectives. The guide is provided in S1 Appendix. A semi-structured approach was taken so that participants would be able to lead the discussion and outline their views with minimal restrictions, whilst being guided to stay on the relevant topics. Since a constant comparative method was utilised, any unanticipated topics were explored further and incorporated into the topic guide for the next participant to ensure that the data collected was as in-depth as possible.

Interviews ranged from 44 to 83 minutes, with an average interview time of 62 minutes. Since the participants were expected to have expert knowledge in the field, the duration of the interviews allowed them to explore various topics in detail.

All the participants gave consent for the interviews to be audio-recorded using an encrypted device and notes were taken during the interview to provide more context to the interviews.

#### Data analysis

The interviews were transcribed verbatim using the audio-recording and were analysed using QSR-Nvivo 12 Software by the lead author, KT. The data was analysed with an inductive thematic analysis approach, using the method proposed by Braun and Clarke [21]. Transcripts were initially coded and concepts were generated from the clustering of codes. Emerging concepts were then organised into themes. Transcripts were reviewed several times in order to improve the coding and refine the emerging concepts. A sample of the coded transcripts were also analytically triangulated by author, TM. A brief of the coding and the summary of the coding framework has been provided In S2 Fig and S2 Table respectively.

## Results

The participant demographic that this study aimed to include were primarily those running the NGOs, such as programme directors, managers, or regional technical advisors. A summary of the participant demographics is provided in S1 Table. Eighteen organisations were identified to potentially suit the study criteria and seven organisations participated in the study. The remaining organisations did not participate in the study for the following reasons: seven did not respond; two refused to participate and two were not able to be interviewed due to time or technical difficulties. No participants were excluded due to language skills, as they all had working levels of English. However, on snowball sampling, the majority of the participants reported that there were only a few organisations implementing trachoma interventions in Tanzania at the time. The number of interviews was therefore deemed representative of the current key stakeholders, especially since data saturation was reached. Although this study set out to understand participants’ experiences with marginalised communities in general, the responses given by participants were focused heavily on Maasai communities. This is likely due to the fact that the endemic areas are populated mainly with Maasai populations, and thus NGOs targeting these areas are currently facing problems adjusting to their unique livelihood and cultural background. This explains why the participants yielded responses primarily focused on the Maasai, despite the study looking at marginalised groups as a whole.

The study found four key themes affecting trachoma intervention implementation: 1) contextual factors; 2) trachoma-specific factors; 3) decision-making factors and 4) the effect of multiple actors.

### 1. Contextual factors affecting trachoma interventions implementation

This theme explores the contextual factors of the marginalized communities identified by NGOs when implementing trachoma interventions, including the key social, economic and political factors.

#### Poor environmental conditions

The majority of participants emphasised the shortage of water in trachoma-endemic areas in Tanzania and recognised that poor or limited infrastructure was an important factor contributing to the shortage of water.

*Because it’s very dry*, *it can’t be someone putting boreholes in the whole village*, *it’s quite impossible*. *[P6*]

Most participants described poor levels of personal hygiene in these marginalised communities as a direct result of water shortage. Several participants recounted that members of these communities were unable to hand-wash or maintain good personal hygiene, despite having been educated during behavioural change trachoma interventions, due to the lack of water infrastructure.

*… educating pupils about hand-washing*, *good sanitation and hygiene*, *but there is no water*, *so they can’t practice practically*. *[P8*]

Some participants pointed out that the absence of latrines or their lack of use resulted in open defecation within the community, providing an environment favourable to the spread and reinfection of trachoma due to the breeding of flies in faeces. It was also outlined that even if latrines were present, open defecation in the community may persist due to habit and reluctance to change their behaviour, contributing to poor environmental cleanliness.

*The sanitation is low already*, *so which means [it] is a component of those flies which are breeding in human faeces*. *[P12*]

*Because building toilets in Maasai communities is one thing*, *but making them to use them is another challenge*. *[P6*]

Most participants outlined that the priority of cattle and the proximity to which they live with the Maasai people created a similar problem to that of open defecation, but with animal faeces. The presence of both human and animal faeces, and poor environmental cleanliness reduces the effectiveness of interventions due to reinfections. A few interviewees also noted that the presence of cattle enhanced the effect of water shortage on personal hygiene, because providing the cattle with water appeared to be regarded as more important than using water for personal cleanliness.

*So until they separate those living quarters [with cattle] … those flies will be*, *will continue to be around … what we are saying is that there is a high prevalence of infection*, *of disease*, *there is all the things that can possibly contribute to transmission*. *[P12*]

#### Traditional lifestyles

The majority of participants described the Maasai upholding their traditional lifestyles, including human population movement. The regular movement of these communities resulted in potential patients being difficult to find and are often missed.

#### Logistical difficulties

All the participants believed that the logistical difficulties as a result of the traditional way of life meant that programmes were not properly implemented in the target populations.

*We think in some villages*, *which I’ve just said*, *the difficult to reach areas*, *maybe they don’t go to every house*. *[P11*]

#### Lack of trust

Most of the participants described that their programme was often received with some scepticism from patients due to the programme being offered by people coming from outside their community. Several participants noted that this was in part due to the language barrier between the field staff and the Maasai communities, as many of the Maasai only speak Maa and not Swahili.

*Language barrier*, *again*, *as I said*, *I told you Maasai don’t necessarily believe someone who’s coming from outside the community*. *[P6*]

#### Community values

Most participants emphasised that hierarchy within the communities is very prevalent and there are different stratifications of the community based on age, sex and status. The stratifications must be respected and there is an expectation that recommendations made by those higher in the community hierarchy are to be followed. The community hierarchy can therefore either facilitate or impede programme implementation, depending on the outlook and influence of the influencer.

*It’s the specific people in the Maasai communities that are sort of the elders*, *the respected people*. *If you don’t get their buy in*, *if you don’t invite them to the sensitisation*, *people aren’t even going to go to your camp at all*. *[P10*]

Some participants felt that the community hierarchy extends to traditional healers and their health beliefs to some extent. Many of them are respected members of the community and therefore members of the community trust them to treat their eye diseases.

*They believed that these traditional healers can help them… they can heal other problems*, *but not trachoma*. *[P11*]

Participants remarked that these healers do not tend to compete with the treatments that their programme offers. They found that traditional healers are often accepting of having healthcare alternatives for their community members, even if it is provided by someone outside their community.

*I think they think of themselves as an alternative treatment for someone who can’t afford or doesn’t want or doesn’t believe in the modern medicine*, *but I don’t think they actively discourage people from getting modern treatments*. *[P10*]

#### Economic context affecting implementation

In regard to the implementation of the programme, four participants highlighted that the programmes are provided free of charge to the communities. As a result, the financial status of the community was not considered a barrier.

Although the programme itself is covered in terms of cost, some of the participants described that there may be some additional costs for the patients seeking treatments for surgery, such as cost of travel to reach the treatments, which acts as a barrier.

*But within that*, *there are many things which are being found to contribute to the low uptake or refusal for surgery and there could be simple things*, *for example*, *people do not have money to travel to their surgeries*. *[P12*]

#### Political landscape

Most participants acknowledged the benefit of having the involvement of the government in trachoma interventions and appreciated the support they received, which facilitates the intervention. Many of the participants had the impression that the government felt that trachoma is a priority on a public health level.

*Through this of course the Tanzanian government has worked very hard with*, *together with the partners to bring down the level of trachoma*, *the disease*. *[P12*]

The political will of the government at the district level was important when it came to how rapidly the programme could be implemented. Additionally, whether a programme can be implemented or not is dependent on the political drive to carry out such changes.

*We see that political people plays major roles in trying*. *So if a politician works together with these people [clicks fingers]*, *things really works very well*. *[P11*]

Despite the best intentions of the government, participants noted that there are still inefficiencies in the implementation by the government. The lack of government resources and funding to carry out a complete programme was a frequently mentioned issue, and resulted in people in trachoma endemic communities being missed or not reached.

*I’m concerned about us trusting that embedding things in the national government is going to solve all our problems*, *I don’t believe that to be the case*. *[P10*]

Several participants noted that case-finders, who find members of the community with trachoma on behalf of the implementing partners, were not being paid much, if at all. Participants found that it often disincentives high quality work.

*Those village level people need to be paid fairly for their efforts*…*I mean no wonder they’re not filling in the registers [P10*]

### 2. Trachoma-specific factors affecting trachoma interventions implementation

This theme explores how factors specific to trachoma, either the disease itself, the beliefs of the people on the disease and its treatments, affect the implementation of trachoma interventions.

#### Knowledge and understanding of the disease and its treatments

Participants reported that there is some awareness of trachoma, owing to the historical presence of trachoma within the communities.

*Remember trachoma is been living with them*, *they’ve been living with trachoma for years*, *before it started modern treatment*. *[P6*]

Several of the participants described patients epilating their eyelashes in order to alleviate pain and prevent blindness. However, this does not necessarily mean that the Maasai make a link between inverted eyelashes and trachoma. As trachoma has been prevalent in their communities historically, it is assumed the inversion of lashes is a normal part of the ageing process.

*They make their own little tweezers out of metal and hang them around their necks*. *Trachoma’s not*, *it’s actually probably not making as many Maasai people blind as it is other people*. *[P10*]

However, generally most participants found that the knowledge and understanding of trachoma is often poor. The most misunderstood aspect of the disease was the causes of the diseases. More than half the participants outlined that patients did not recognise that the disease is an infection spread by flies and believed in various causes such as trachoma being hereditary or witchcraft. These misunderstandings render implementation of the programme more challenging.

Participants also established that patients often had misbeliefs regarding the treatments provided by specific programmes, which acted as a deterrent in uptake of treatment. These beliefs often arose due to seeing others have bad experiences, such as side effects or confusion with other conditions. Rumours within the communities also serve as a barrier to accepting treatment, which result in propagating misbeliefs, especially when they conflict with the knowledge being advocated by programme implementers. A few participants elaborated that some patients, after undergoing trichiasis surgery, developed blindness due to a different ophthalmic condition. However, the patients incorrectly believed that the loss of vision was due to trichiasis surgery.

*If you offer surgery without telling them that you also have cataract*, *or glaucoma*, *at some point*, *they will end up being blind*, *due to cataract or glaucoma*, *so they tend to associate the [trichiasis] surgery with the vision loss [P7*]

Several participants described a disconnect between knowledge and action, due to the lack of available facilities and infrastructure to carry out good hygiene and maintain a clean environment. Even if the members of the community have sufficient knowledge to carry out the necessary behavioural changes to reduce the spread of trachoma, they cannot change their practices due to the lack of available resources.

#### The location of the disease

With trachoma being an ocular disease, the majority of participants mentioned that the eye is a particularly sensitive area. Some participants described that patients from marginalised or Maasai communities were more reluctant to receive surgery in the fear that the treatment will render them blind, as being able to see facilitates their lives.

*Maasai people depend on being able to see*, *because they’re pastoralists*. *They need to be out there*, *walking their animals*, *they’re not living in a house*. *[P10*]

This effect is compounded by the fear of surgery in general. As many of the Maasai people are unaccustomed to or scared of the idea of surgery, having an operation on a sensitive area of the body presents some difficulties when convincing patients to receive treatment.

*I think generally*, *in the African setting*, *the moment you say that you are going to operate somebody*, *[laughs]*, *the moment you are offering surgery*, *everybody*, *on almost anything*, *then you can imagine now*, *even with the eye*, *then it becomes even more challenging*. *[P12*]

#### The progression of the disease

Many participants remarked that community members did not understand that trachomatous inflammation and trachomatous trichiasis was caused by the same disease at different phases of disease progression.

*By the time they develop trichiasis*, *which is the in-turning of eyelashes*, *they don’t have infection*. *The infection was some years ago*. *So by the time they develop trichiasis*, *they don’t have infection*. *So they think they are fine*. *[P11*]

This lack of understanding of the disease progression has negative implications on the implementation of the programme. One participant elaborated that some patients no longer trusted azithromycin because their disease had progressed to trichiasis despite having been given MDA previously.

### 3. Factors that affect decision making in the implementation of trachoma interventions

This theme explores the considerations that are made when deciding what and how the organisation implements the trachoma interventions. This includes whether the intervention tailors to the community needs, whether it is financially feasible and whether it helps improve the quality of the programme.

#### Tailoring to community needs

All the participants recognised the importance of tailoring programmes for specific communities. This requires good cultural understanding on the part of the implementers in order to improve uptake of interventions.

*So we are trying to make sure that we offer service*, *but also*, *they [members of the community] should be comfortable that the service doesn’t interfere too much with the culture [P6*]

Several organisations also recognised that different communities have slightly different needs, and this results in making decisions to adjust each programme accordingly. Therefore, having flexibility within the organisation is a facilitator for successful implementation of programmes.

#### Improving programme quality

Most participants outlined that having quality checks are crucial to making decisions for an effective programme. The quality checks allowed for progress to be monitored and therefore improvements could then be made if the programme is found to be poorly implemented or negatively affecting patient care.

*From the auditing*, *we confirm that two of the surgeons were not doing good surgery*. *So they were*, *they were mentored but it seems they were not improving and we asked them to stop doing surgery and the number of surgical failures dropped down*. *[P7*]

All the participants felt that the ability to identify problems and reflect on them was an important asset contributing to decision making in an organisation. It was also noted that much of this reflection is done alongside other organisations or the government.

*Part of the annual planning identifies the gaps*, *ok*, *so when you get new partners and the government is able to suggest where it is that they would add value*. *[P12*]

All the participants mentioned that research improves the programme quality as well, and several of the participants elaborated that the findings from research have been used to develop manuals that guide programme implementation.

*The WHO and other organisations assign [us] to play a role for capacity building and also for research*, *and developing the manuals*. *[P6*]

#### Financial feasibility

Nearly all the participants affirmed that the amount of available funds dictates the activities of the programme, and adjustments are sometimes needed in order for the programme to fall within budget.

*Funding is within the budget*, *you have been given a ceiling*. *And you have to plan things according to that ceiling*. *If I get more funds*, *of course*, *I will design other things of going to do*, *maybe I can increase the number of going to the houses*. *[P11*]

There is a discrepancy in the funds directed towards the S and A compared to F and E. Organisations tackling F and E interventions elaborate more on not having enough funding to do a well-rounded or comprehensive, district-wide programme, compared to the organisations focusing on S and A interventions.

*Yeah*, *F and E is very challenging and then there are*, *as I mention before*, *even among the donors themselves*, *they are more willing to support A and S*, *than F and E*. *[P6*]

This reflects the quality and quantity of the F and E programmes. Budget and finances are therefore a key factor in the decision-making aspects of a programme and participants recognise that there is an opportunity cost to every programme.

### 4. Multiple actors affecting trachoma interventions implementation

This theme explores how having multiple actors supporting trachoma control in Tanzania influences implementation of individual interventions.

#### Cooperation between organisations

Most participants discussed a sense of cooperation between the organisations working on trachoma control in Tanzania. Several participants elaborated that the cooperation is particularly enforced because each partner needs to put in their fair share of work to eliminate trachoma, since no two organisations has worked in the same district for the same interventions.

*NGOs will bring in their funds*, *but also their experience*, *their expertise*, *but also they*, *the government will also bring in the staff and the other resources*. *So that’s the kind of partnership we want*. *If one of them breaks*, *then definitely the programme will not work*. *[P6*]

A few participants felt that the cooperation among the different actors would provide opportunities in increasing the efficiency of the programmes. It was recognised that some parts of the programme could be done concurrently instead of as separate programmes.

*What we are doing is how can we collaborate*, *to take advantage of*, *for instance…if they’re doing antibiotic mass drug administration*, *then that’s an opportunity for us to screen [for TT]*. *[P6*]

It was also identified that each organisation has its own areas of expertise, and the way that the districts are split up between them optimises their programme to their abilities. The organisations therefore appear to rely on one another so that each district receives components of the SAFE strategy.

*That’s outside of our scope*, *it’s also outside of our forte*, *we look to partner with organisations that have country offices and boots on the ground*, *who know the population and have hired nationals from that country who are very familiar with the region and the politics and the geography [P7*]

#### Learning from other programmes

Most participants stressed their appreciation of having quarterly meetings with multiple actors in order to discuss progress and programmes. The participants felt learning from other programmes provided them with additional experience in the field and exposed them to more effective solutions. Several participants also pointed out that learning of and comparing the progress between trachoma interventions by different organisations allowed them to reflect as to whether their programme was headed in the right direction.

*Every three months*, *we sit together and do what we call programme review meeting*. *By doing that*, *we are getting experience of what others do… from what I know*, *we are*, *our NGO is doing well*. *[P11*]

Several participants explained that there were more training opportunities for staff as a result of multiple actors in trachoma interventions. Type of training described varied between participants and some described sending staff to other programmes for observation, whilst others described attending workshops hosted by another implementor.

#### Variability in programme implementation

More than half participants outlined that having multiple actors working in districts in a non-overlapping fashion meant that there was variability in the success of programmes among districts.

*I think some partners are*, *yeah*, *a couple partners work very quickly*, *find a lot of patients*, *seem very efficient*. *Some partners seem less efficient*, *find fewer patients*. *[P10*]

Participants noted that there was also variability between different components of the SAFE strategy, each of which are generally managed by separate organisations. The majority of participants felt that the F and E aspects were somewhat lacking compared to the other two components and the most commonly mentioned reason for this discrepancy was due to less funding. Several participants attributed this to donors being less willing to support behavioural change interventions owing to the lack of measurability of the programmes and therefore could not see any discernible benefit from its implementation.

Even though some of the behavioural change aspects are handled by partners working on trachoma, the majority of participants highlighted that some districts had parts of F and E handled by organisations not necessarily focused on trachoma specifically, but on general hygiene and sanitation. A few participants discussed that although these general programmes did have some benefit, they may not necessarily be in-line with the most ideal trachoma control programme.

*Where they think it is important for them to supply water is not necessarily what where we think from a trachoma perspective*. *Because they have the other plans*. *[P6*]

*And that no point*, *in any of their manuals*, *does it mention wash your face a couple times a day too*. *So that’s a huge gap*. *[P10*]

## Discussion

Many of the NGOs felt that the understanding and treatment of the disease was often poor, which acted as a barrier to implementation. NGOs felt that this poor understanding contributed to the fear of surgery within the Maasai. However, it is important to understand the basis of that fear, rather than using the general concept of ‘fear’ as a blanket statement to explain refusals in a simplistic manner, in order to be able to help the community overcome their fear of the treatment. Within Maasai communities, the fear was often not of the surgery itself, but rather a fear of the consequences associated with surgery, such as fear of long recovery times, fear of pain after surgery or fear of blindness after surgery [22]. Additionally, the poor understanding of trachoma as a disease, where active trachoma and trichiasis were not associated with each other owing to the lengthy time-lag contributed further to difficulties with trachoma interventions. Within the Maasai culture, healthcare decisions are often made by a family unit, rather than solely dependent on the wishes of the individual [23]. This therefore highlights the need for improved counselling services to explain the practical aspects of surgery or any concerns and should be done with family members that are of age. This is particularly relevant if the person affected with trichiasis is female, since males are the predominant decision makers in a domestic unit [24]. The majority of counselling should done by the surgical outreach team early on and there should be at least one person on the team dedicated to counselling to render the service more focused, to reduce the likelihood of counselling being evaded, and to instill a sense of responsibility in the counsellor to ensure that potential patients and their families are well-informed. Ideally, this role should be undertaken by someone to whom the other members of the community can relate and who speaks the local language proficiently. Asking members of the community who previously have had positive experiences with surgery to become ambassadors may be an effective way of advocating surgery.

One study looking at the Maasai perspective of trachoma found that the Maasai recognised that western medicine was effective [25]. This was contrasted by the findings of this study, where NGOs perceived that the Maasai were skeptical of, and therefore reluctant to receive western medicine. This may be due to miscommunication on both fronts and the community’s poor understanding of NGOs’ goals [25]. Because many of the workers that implement trachoma interventions are not necessarily Maasai, implicit preconceptions of the marginalised groups may have been made, not out of malicious intent, but derived unconsciously. While pragmatic issues during interventions certainly exist, such as language barriers or travelling to remote areas, these preconceptions may further negatively impact the intervention provided, by affecting judgement and altering non-verbal behaviour subconsciously [26], and impact the receptivity and acceptability of the interventions to marginalised populations.

A systematic review demonstrated that the levels of implicit bias exhibited by healthcare professionals are similar to that of the general population [26], which may be mirrored when implementing trachoma interventions. Seeing as the Maasai are relatively disadvantaged as an ethnic group in terms of health compared to other surrounding ethnic minority groups [27], it is important to minimise “corrosive disadvantage”. This is where an already vulnerable group is further disadvantaged as a result of current disadvantages [28]. A study in Tanzania, Kenya and Namibia assessing stigma exhibited by tuberculosis health care workers on patients with HIV established that teams that received regular supervision as well as training to better understand patient context and ways to recognise implicit bias improved care [29]. Having regular training sessions may therefore help health care or educational personnel to recognise and reflect upon any pre-existing ideas or misconceptions and improve the dynamic between the implementing teams and the community.

NGOs described that simply providing behavioural change interventions is not sufficient, in both school-based and community-based interventions, if there are no facilities to actually carry out the change. Partners recounted various difficulties in funding, logistics, time and manpower as the main obstacles for erecting sufficient infrastructure, such as latrines or boreholes, for good hygiene practices. However, there is a certain degree of cognitive dissonance with this mode of reasoning, as it fails to take into account the biosocial needs of the community. In practice, even in the instances where such infrastructure has been built, explanations for why or how this equipment should be used is not always provided, giving little rationale for members of the local community to utilise them. Additionally, focusing too much on an immobile option to improve sanitation overlooks the pastoralist nature of the Maasai. These issues have been reflected in the literature, as there have been instances where simply increasing the number of latrines or other facilities does not improve hygiene outcomes, even when provided synergistically to a behavioural change water and sanitation programme (WASH)[30]. A review by *Kamara et Al*. showed that when behavioural changes do occur, it has been associated with improved hygiene outcomes, however, actually sustaining these behavioural changes in the long-term has repeatedly proven a challenge [31]. The pinnacle is that it does not appear that the lack of infrastructure is the main barrier to partners for low levels of sanitation in the communities. Ultimately, it is more important to find other options to better suit the culture of the Maasai, as well as determine superior ways of instilling and maintaining behavioural change for good hygiene practices through education, and further research would be needed to identify the best method to do so.

In Tanzania, the current approach for behaviour change regarding WASH uses community-led total sanitation (CTLS) and is implanted by the governmental sanitation programme. This approach focuses on evoking strong emotions to trigger the community to take responsibility for the cleanliness of their environment [32]. Although CLTS has been globally recognised as a relatively inexpensive form of sanitation programme and has been found to reduce the prevalence of open defecation in some communities, the widespread benefits that have been reported are often overly optimistic [33]. However, the very nature of the programme, in using emotions such as shame or disgust, may result in the community conforming to standards that may not necessarily be compatible with local culture or the socio-economic and political aspects of the local community [32–34]. There has also been discussion as to whether this type of programme propagates further stigmatisation of marginalised groups [35]. Such approaches therefore may not be the most appropriate initiative for marginalised communities, particularly in groups like the Maasai, who hold their cultural and traditional beliefs very strongly.

NGOs and the government should collaborate to gain further insight in the needs and the dynamics of the local communities, either through empirical experience or further research, in order to improve the acceptability of the national sanitation programmes in these marginalised communities. There should either be a change in the approach that the current CLTS programme uses, where the local context can often be neglected due to the lack of tailoring in the programme, or a separate guideline conscious of the context in these communities should be developed. Alternatively, focusing on other types of sanitation programmes, such as school-based interventions, may also contribute to more successful outcomes. School-based interventions have been described to be somewhat effective and children that attend school have a greater awareness of general sanitation [36]. One study found that in rural Tanzania, with a predominant Maasai community, mothers with a higher level of education are significantly more likely to have better hygiene practices and their children were less likely to suffer from diarrhoeal illness [37]. It has also been repeatedly found that households with better-educated caregivers maintain a higher standard of sanitation in Sub-Saharan Africa [38]. This suggests that the effect of school-based interventions may instil better hygiene habits in an intergenerational manner.

Multitudes of healthcare models and determinants of health have been developed in order to offer guidance in the programme design, but in reality, only a minority use a model as a basis for their intervention, especially in resource-limited settings [39,40]. Considering the strong cultural beliefs and the nature of the Maasai, the design and implementation of a behavioural change intervention would be most effective if underpinned by a behavioural model that considers the contextual, psychosocial, economic and political context. Having sufficient community engagement in both the planning and implementing phase should also be regarded as a priority, in order to tailor programmes and minimise issues related to the perception of power imbalance and control between the outside “implementers” and the “community”[41]. This empowers the members of the community to advocate for their own hygiene and sanitation. By extension, this would also reduce any unconscious preconceptions and implicit bias from an external implementor.

Within the community, the majority of patient recruitment for trachoma interventions are done by community health volunteers. Although there often is an expectation for individuals to take responsibility for the health of their community, and there is encouragement to recognise that “these are their issues and they need to fix it themselves”, the lack of financial remuneration may result in lower quality recruitment and other aspects of intervention [42]. In the Morogoro region, there was a high spirit to volunteer and improve community health, but the inability to generate enough income to survive, owing to the volunteer nature of the community health worker as well as the opportunity cost of time, served as a deterrent and resulted to less time devoted to community health activities [25,43]. As a result, not only are there difficulties on the part of those receiving trachoma interventions as a result of economic context, but there are also difficulties in terms of members of the community advocating trachoma intervention as well. NGOs should consider providing transparent and fair compensation for the time provided by community volunteers [25]. It is understandable, however, that from a practical perspective, NGOs may not have sufficient funds for this and therefore would require additional input from the government, alongside a strong political will to carry out significant changes in the current system.

The economic context faced by NGOs is also relevant in implementing their programmes. One study in Australia described an increase in prevalence in trachoma when funding, and therefore trachoma intervention, was delayed [44]. A systematic review determined that, in low- and middle-income countries, financial limitation was the main barrier to exchange of health information, which is relevant to the F and E components of the SAFE strategy [45]. This study therefore builds upon the concept of the lack of funding, as multiple participants have described a disparity between the funding towards F and E programmes compared to S and A. For Tanzania, this disparity may not be because of the lack of available funds *per se*, but because of the poor perception of the effectiveness of F and E programmes by donors, which drives financial constraints. Therefore, changing the attitudes of donors regarding the SAFE strategy may be an essential part in the elimination of trachoma in marginalised communities.

NGOs described considerations of overall programme quality and needs of the specific community when making programmatic decisions. On an organisational level, both quality checks and research inform decisions within the organisation by providing feedback on the programme and guides supervision. The benefit of receiving and incorporating feedback into healthcare programmes has also been described in other studies on healthcare improvement in lower income countries [39–41]. Increased supervision of field teams can be used as a form of quality check by NGOs that should be done regularly but randomly, so as to ascertain a more accurate picture of the effectiveness of interventions. For MDA use in NTDs, several studies have highlighted a discrepancy between actual drug intake and the amount of drug provided [46–48]. Often, it has been found to be a result of poor compliance and a lack of understanding of the treatment, which are consistent with the barriers depicted by NGOs in this study. However, none of the NGOs discussed the possible inaccuracies associated with the reporting of MDA figures as a reason for the persistence of the disease. The current method of reporting MDA coverage is based on how many pills are given out, which fails to consider whether MDA is actually received or consumed by the target population [25,46–48]. As a result, the coverage figures may not mirror reality. There is no doubt that MDA is useful in the elimination of trachoma, but in order to fully eliminate the disease, particularly within hard to reach communities in Tanzania, reporting and feedback must be accurate to inform and improve the programme. Hence, there is a need for sufficient quality checks in the implementation of programmes as well as an increased stringency on the methods used to report MDA coverage.

In order to improve programme quality, by both improved intervention provision, improved communication or assessing inefficiencies, NGOs can consider incorporating the use of technology in interventions, which has been explored in other Sub-Saharan countries. Despite the fact that the Maasai live traditionally, there has been a recent increase in the use of modern technology such as mobile phones in communities [49]. Many of the Maasai communities are already using technology for business as well as getting in touch with members of other bomas [50].

In Chad, mobile technology has been used as a low-cost method in order to better understand the behaviour of pastoralists, including migration patterns [51]. Surveys were also being done with the use of technology and were found to be feasible [51]. Considering that NGOs describe that the remote nature and the mobile lifestyle adopted by the Maasai were barriers to trachoma intervention, it may be worthwhile exploring whether this technology in the future could be used to facilitate locating groups in remote areas. It may also provide opportunities for community members to provide additional feedback to improve programmes. There have already been instances where successful mobile health interventions in Ghana and Nigeria have been developed with the backing of the national government, though there is no doubt that a strong political drive has played an important role in rolling out these interventions [52]. However, as the current literature on incorporating such technologies in a resource-limited setting with pastoralist communities are limited, further research on understanding facilitators and barriers of using newer technology, as well as studies to determine the most effective type of mobile intervention would provide valuable information for the development of a reformed programme.

The presence of multiple actors implementing trachoma interventions was a facilitator in reaching the goal of trachoma elimination. The Global Trachoma Mapping Project described that collaboration between different partners and the government is essential in order to carry out trachoma elimination [53]. The findings of this study emphasised the fact that the current system between partners in Tanzania appears to work well and is supported by epidemiological studies demonstrating a significant drop in trachoma prevalence [53]. However, collaboration between stakeholders, either in the field or through joint training programmes, should be strengthened even more to enhance the effectiveness of interventions in marginalised communities and to reduce intervention variability between districts. Quality checks between organisations should be standardised to better measure and improve any variability.

Although the sample size of the study was relatively small, there is only a limited number of NGOs implementing trachoma intervention programs in Tanzania. As a result, the majority of the key stakeholders were included in the study and data saturation was reached. This in turn suggests that the data is somewhat generalisable to the implementation of trachoma programmes in Tanzania.

One of the limitations of this study was the risk of social acceptability bias. The nature of the questions asked in the study may have resulted in participants focusing more on the issues that were external to their organizations, rather than factors within their own organizations that were barriers to programme implementation. As a result, much of the context provided by these organizations may not be a wholly accurate representation of the beliefs and behaviours of Maasai or other marginalised communities in Tanzania. Despite this, understanding the perspectives of these organisations is still valuable to improving trachoma interventions.

## Conclusion

This is the first study looking at the perspectives of NGOs on implementing trachoma programmes in marginalised communities in Tanzania and specifically Maasai communities. Overall, difficulties experienced by partners in providing health education and sustaining behavioural change has limited the success in reducing the prevalence of trachoma. These changes need to be finely tailored to the needs and culture of the target community to ensure that interventions are acceptable and sustainable. Without significant improvements in community education, in counselling, understanding of the disease as well as basic hygiene measures, programmes may continue to face poor uptake. Organisations should also ensure that their programmes have ways to incentivise high-quality work as well as have appropriate procedures to monitor the programme quality and ensure the accountability of staff. Incorporating the use of mobile technology in the future may have some potential to improve programme quality and reduce some of the barriers faced by NGOs, though there is a need for further research to determine the feasibility and logistics. If there is a strong political will from both the district and national government to make these changes, eliminating trachoma in these marginalised communities may be realised sooner rather than later. Future research should focus on improving understanding of perceptions of marginalized communities and their behaviours in relation to trachoma, as it would provide insight on how NGOs can better tailor their future programmes to suit these communities.

## Supporting information

S1 Fig Core Themes and Subthemes Represented Schematically. (DOCX)

S2 Fig Coding Briefing. (DOCX)

S1 Table Participant Demographic. (DOCX)

S2 Table Summary of Coding Framework. (DOCX)

S1 Appendix Topic Guide. (DOCX)
