# Supplementary material for: Implementing trachoma control programmes in marginalised populations in Tanzania: A qualitative study exploring the experiences and perspectives of key stakeholders
Source: PLoS Negl Trop Dis. 2021 Sep 10;15(9):e0009727. doi: 10.1371/journal.pntd.0009727 (PMC8432809; doi:10.1371/journal.pntd.0009727)
Supplement: S1 Fig — (DOCX) [file pntd.0009727.s001.docx]

| **Experience and Perceptions of TCP implementation by NGOs** | **Core Themes** | **Subthemes** |
| --- | --- | --- |
|  | Contextual factors | Social Context |
|  |  | Economic Context |
|  |  | Political Landscape |
|  | Trachoma-specific Factors | Anatomical location of disease |
|  |  | Disease progression |
|  | Decision-making factors | Tailoring to community needs |
|  |  | Improving programme quality |
|  |  | Financial feasibility |
|  | The effect of multiple actors | Cooperation between organisations |
|  |  | Learning from other programmes |
|  |  | Variability in programme implementation |

**S1 Fig: Core Themes and Subthemes Represented Schematically**
