# Supplementary material for: Implementing trachoma control programmes in marginalised populations in Tanzania: A qualitative study exploring the experiences and perspectives of key stakeholders
Source: PLoS Negl Trop Dis. 2021 Sep 10;15(9):e0009727. doi: 10.1371/journal.pntd.0009727 (PMC8432809; doi:10.1371/journal.pntd.0009727)
Supplement: S2 Fig — (DOCX) [file pntd.0009727.s002.docx]

**S2 Fig: Coding Briefing**

| **Subthemes** | **Codes** | **Subcodes** |
| --- | --- | --- |
| **Social Context** | Environmental Issues | Cattle as a health issue |
|  |  | Lack of use of toilets - open defecation |
|  |  | Logistical Issues |
|  |  | Personal Hygiene |
|  |  | Poor Infrastructure |
|  |  | Priority of cattle |
|  |  | Not reaching all patients |
|  |  | Water shortage |
|  |  | Flies |
|  | Education | Expectations |
|  |  | General education |
|  |  | History of Trachoma |
|  |  | Incorrect health beliefs on treatment |
|  |  | Knowledge of trachoma |
|  |  | Knowledge vs Action |
|  |  | Literacy |
|  |  | Poor knowledge of trachoma |
|  |  | Trachoma is normal |
|  |  | Day vs boarding school |
|  | Lifestyle | Personal Hygiene |
|  |  | Nomadic lifestyle |
|  |  | Priority of cattle |
|  |  | Lack of use of toilets - open defecation |
|  |  | Logistical Issues |
|  | Community Values | Spiritual connection |
|  |  | Local health beliefs and treatment |
|  |  | Language |
|  |  | Scepticism of outsiders |
|  |  | Children vs adults |
|  |  | Community Hierarchy |
|  |  | Difference in Sexes |
|  |  | Spiritual connection |
| **Economic Context** | Economic Status of the Community | Cattle as a health issue |
|  |  | Lack of resources |
|  |  | Knowledge vs Action |
|  |  | Lack of other eye care |
|  |  | Trachoma as disease of poverty |
|  |  | Logistical Issues |
|  |  | Economic Status |
|  |  | Lack of use of toilets - open defecation |
|  | Economic context of NGOs | Different agendas |
|  |  | Lack of resources specific to ophthalmology |
|  |  | Logistical Issues |
|  |  | Poor Infrastructure |
|  |  | Cost-to-benefit |
|  |  | Cutting cost |
|  |  | Lack of resources |
|  |  | Budget |
| **Political Landscape** | Governmental Context | Ineffective government context |
|  |  | Supportive government context |
|  |  | Political conflicts |
|  |  | Politics affecting optimal programmes |
|  |  | Political facilitators |
|  |  | Will to carry out programme |
|  |  | Poor work culture |
|  |  | Trachoma is a priority |
|  |  | Following international guidelines |
|  |  | Government decision-making |
|  |  | Government Involvement |
| **Knowledge and Understanding of the Disease and its Treatments** | Perceptions | History of Trachoma |
|  |  | Blood in Maasai culture |
|  |  | Eye is a sensitive area |
|  |  | Belief of the consequences of trachoma |
|  |  | Belief that trachoma is hereditary |
|  |  | Trachoma is normal |
|  |  | Double standard (only TF or TT is being treated) |
|  |  | Importance of Sight |
|  |  | Trachoma not seen as priority |
|  |  | Poor knowledge of trachoma |
|  | Behaviours | Too late |
|  |  | Surgery is necessary |
|  |  | Confusion between TF and TT |
|  |  | Double standard (only TF or TT is being treated) |
|  |  | Local health beliefs and treatment |
| **Anatomical Location of Disease** | Eye disease | Confusion between TF and TT |
|  |  | Importance of Sight |
|  |  | Eye is a sensitive area |
|  |  | Pain |
| **Disease Progression** | Disease Progression | Reinfections |
|  |  | Recurrence and persistence |
|  |  | Belief that trachoma is hereditary |
|  |  | Trachoma is normal |
|  |  | Double standard (only TF or TT is being treated) |
|  |  | Poor knowledge of trachoma |
|  |  | Confusion between TF and TT |
|  |  | Trachoma not seen as a priority |
|  |  | Local health beliefs and treatment |
| **Tailoring to Community Needs** | Tailoring to Community | Incentivisation |
|  |  | Familiarity with the programme |
|  |  | Financial Barriers |
|  |  | Knowledge vs Action |
|  |  | Compromise |
|  |  | Cultural understanding |
|  |  | Behavioural Understanding |
|  |  | Language |
|  |  | Learning from other programmes |
|  |  | Less experience |
|  |  | Local health beliefs and treatment |
|  |  | Long term sustainability |
|  |  | Merging programmes |
|  |  | Flexibility |
|  |  | Planning ahead |
|  |  | Past experiences |
| **Improving Programme Quality** | Quality Checks | Consistency and standardisation |
|  |  | Familiarity with the programme |
|  |  | Identifying problems |
|  |  | Incentivisation |
|  |  | Quality Checks |
|  | Staff Factors | Programme Hierarchy |
|  |  | Rapport building |
|  |  | Supervision |
|  |  | Support programme |
|  |  | Teamwork |
|  |  | Training |
|  |  | Update knowledge |
|  |  | Will to carry out programme |
|  |  | Flexibility |
|  | Organisational | Learning from other programmes |
|  |  | Avoiding Delay |
|  |  | Awareness of what is seen may not reflect reality |
|  |  | Merging programmes |
|  |  | Finding opportunities |
|  |  | Flexibility |
|  |  | Planning ahead |
|  |  | Learning from other programmes |
| **Financial Feasibility** | Financial Feasibility | Cost-to-benefit |
|  |  | Cutting cost |
|  |  | Lack of resources |
|  |  | Budget |
| **Cooperation** | Facilitators to the Programme | Research to improve programmes |
|  |  | Supervision |
|  |  | Quality checks |
|  |  | Training |
|  |  | Teamwork |
|  |  | Communication |
|  |  | Cooperation with government |
|  | Learning from other Programmes | Cooperation with other NGOs |
|  |  | Learning from other programmes |
|  |  | Training |
| **Variability in Programme Implementation** | Barriers to Programmes | Perception that F and E are less important |
|  |  | Variability in districts |
|  |  | Variability in NGOs |
|  |  | Different agendas |
|  |  | Multi-sectorial approach |
|  |  | Training |
|  |  | Lack of resources |
|  |  | Measurability of the outcome of the programme |
