# Supplementary material for: Implementing trachoma control programmes in marginalised populations in Tanzania: A qualitative study exploring the experiences and perspectives of key stakeholders
Source: PLoS Negl Trop Dis. 2021 Sep 10;15(9):e0009727. doi: 10.1371/journal.pntd.0009727 (PMC8432809; doi:10.1371/journal.pntd.0009727)
Supplement: S1 Table — (DOCX) [file pntd.0009727.s003.docx]

**S1 Table: Participant Demographic**

| **Participant Number** | **Position in Organisation** | **Aspects of SAFE covered by the organisation** |
| --- | --- | --- |
| 6 | Director | S, occasionally F and E |
| 7 | Programme Manager | S and A |
| 8 | Project Officer | F and E |
| 9 | Assistant Director | A |
| 10 | Director | S, F and E |
| 11 | Programme Manager | S |
| 12 | Regional Technical Advisor | A |
