# Supplementary material for: Implementing trachoma control programmes in marginalised populations in Tanzania: A qualitative study exploring the experiences and perspectives of key stakeholders
Source: PLoS Negl Trop Dis. 2021 Sep 10;15(9):e0009727. doi: 10.1371/journal.pntd.0009727 (PMC8432809; doi:10.1371/journal.pntd.0009727)
Supplement: S2 Table — (DOCX) [file pntd.0009727.s004.docx]

**S2 Table: Summary of Coding Framework**

| **Themes** | **Summary of Codes** | | | | | | |
| --- | --- | --- | --- | --- | --- | --- | --- |
|  | **Participant 1** | **Participant 2** | **Participant 3** | **Participant 4** | **Participant 5** | **Participant 6** | **Participant 7** |
| **Social Context** | Environmental issues, logistical issues, Scepticism of outsiders, incorrect health beliefs | Poor knowledge of trachoma, logistical issues, scepticism of outsiders | Environmental issues, Community Values, Lifestyle, Education | Lifestyle, community values, education, environmental issues | Community values, environmental issues, lifestyle, education | Environmental issues, education, community values, lifestyle | Environmental issues, community values, lifestyle, education |
| **Economic Context** | Logistical issues, poor infrastructure | Logistical issues, lack of other eye care, poor infrastructure | Poor infrastructure, logistical issues, lack of use of toilets | Logistical issues, economic status | Poor infrastructure, logistical issues, lack of use of toilets, different agendas | Logistic issues, poor infrastructure, lack of resources | Poor infrastructure, lack of use of toilets, economic status |
| **Political Landscape** | Will to carry out programme, government support | Supportive government context, will to carry out programme | Politics affecting optimal programmes, supportive government context | Will to carry out programme, politics affecting optimal programme | Will to carry out programme, ineffective government context, government decision-making | Will to carry out programme, political facilitators, supportive government context | Supportive government context, ineffective government context |
| **Knowledge and Understanding of the Disease and its Treatments** | Local health beliefs and treatment, history of trachoma | Poor knowledge of trachoma, Double standard (only TF or TT is being treated), local health behaviour and treatment | Local health beliefs and treatment, F and E vs S and A, poor knowledge of trachoma | Local health beliefs and treatment, poor knowledge of trachoma | Local health beliefs and treatment, trachoma not seen as priority | Local health behaviour, history of trachoma, | Local health behaviour, Poor knowledge of trachoma, |
| **Anatomical Location of Disease** | Importance of sight, eye is a sensitive area | Confusion between TF and TT, importance of sight, eye is a sensitive area | Importance of sight | Confusion between TF and TT | Importance of sight, eye is a sensitive area | Importance of sight, confusion between TF and TT | Importance of sight, eye is a sensitive area |
| **Disease Progression** | Beliefs of the consequences of trachoma, poor knowledge of trachoma | Poor knowledge of trachoma, Double standard (only TF or TT is being treated) | Local health beliefs and treatment, Too late | Reinfections, poor knowledge of trachoma | Recurrence and persistence, Confusion between TF and TT | Recurrence and persistence | Reinfection, recurrence and persistence, Double standard (only TF or TT is being treated) |
| **Tailoring to Community Needs** | Cultural understanding flexibility, local health beliefs | Financial Barriers, familiarity with the programme | Cultural understanding, ownership (of community), Knowledge vs Action | Familiarity with the programme, financial barriers, cultural understanding | Cultural understanding, financial barriers, local health beliefs and treatment | Cultural understanding, flexibility, teamwork | Cultural understanding, local heath beliefs and treatment, long-term sustainability |
| **Improving Programme Quality** | Flexibility, consistency and standardisation, familiarity with programme | Familiarity with the programme, quality checks | Clear organisation, flexibility, programme hierarchy | Familiarity with the programme, quality checks, will to carry out programme | Quality checks, flexibility, consistency and standardisation | Quality checks, flexibility, will to carry out programme | Merging programmes, programme hierarchy, planning ahead |
| **Financial Feasibility** | Lack of resources, cost-to-benefit | Lack of resources | Lack of resources, financial barriers | Financial barriers, financial facilitators, lack of resources | Financial barriers, lack of resources, cutting cost | Financial facilitators, financial barriers, lack of resources | Lack of resources, financial barriers |
| **Cooperation Between Organisations** | Cooperation with outher NGOs, cooperation with government | Training, learning from other programmes, research to improve programmes | Programme Hierarchy, research to improve programmes, cooperation with other NGOs, supportive government | Cooperation with other NGOs, Teamwork, research to improve programmes | Cooperation with other NGOs, training | Quality checks training, cooperation with other NGOs, teamwork | Communication, cooperation with other NGOs |
| **Learning from other Programmes** | Learning from other programmes, training | Learning from other programmes | Learning from other programmes, training | Cooperation with other NGOs | Cooperation with other NGOs, learning from other programmes | Learning from other programmes, training | Cooperation with other NGOs, supervision |
| **Variability in Programme Implementation** | Variability in districts | Variability in districts, perception that F and E are less important than S and A | Variability in districts, perception that F and E are less important than S and A, measurability of the outcome of the programme | Variability in districts, lack of resources, perception that F and E are less important than S and A | Training, perception that F and E are less important than S and A, variability in districts | Will to carry out programme, lack of resources, variability in districts | perception that F and E are less important than S and A, variability in districts, lack of resources |

TT – Trachomatous trichiasis TF – Follicular trachoma A – Antibiotics

F – Facial Cleanliness S – Surgery E – Environmental Change
