# Supplementary material for: Implementing trachoma control programmes in marginalised populations in Tanzania: A qualitative study exploring the experiences and perspectives of key stakeholders
Source: PLoS Negl Trop Dis. 2021 Sep 10;15(9):e0009727. doi: 10.1371/journal.pntd.0009727 (PMC8432809; doi:10.1371/journal.pntd.0009727)
Supplement: S1 Appendix — (DOCX) [file pntd.0009727.s005.docx]

**S1 Appendix: Topic Guide**

| **Objective** | **Sub-topics** | **Questions and Probes** |
| --- | --- | --- |
| Contextual factors influencing the delivery of trachoma control programmes (TCPs) | Social Context | - What is the social context in which your NGO has to work with? - What is the overall awareness of trachoma in the Maasai/marginalised communities? - What are the social factors within these communities that facilitate the implementation TCPs, and how do they do so? - What are the social factors within these communities that make it the implementation of TCPs difficult, and how do they do so? - What is the level of education (knowledge) in these communities on trachoma?   - What is it?   - How to treat it?   - How to prevent it? - Have you ever considered tailoring your programme for these specific communities?   - Give an example? |
|  | Economic Context | - What is the economic context within these marginalised communities? - Do economic barriers affect the uptake of TCPs?   - Do you consider this when you plan your programmes/how have you altered you programme with this in mind? - What is the state of inequality within these communities, and does this affect the delivery of TCPs? |
|  | Larger Context | - How much of a priority is trachoma on a national level? - Why do you think it is (or isn’t) a priority? - What are some of the ongoing aspects in leadership in marginalised communities? - Does this affect the delivery of TCPs, and if so, why? - Do the communities have any political issues within themselves (e.g. in their tribes)?   - Maasai have had issues with land in northern Tanzania, does this affect implementing these programmes? - Has there been any recent changes in terms in the larger context? - If so, how has these changes affected delivery of TCPs? |
| Trachoma-specific factors influencing the delivery of the of the control programmes | Difficulties regarding the disease itself | - Are there any factors about trachoma that makes the delivery of TCPs difficult? - Are trachoma-control programmes more difficult to be implemented compared to programmes for other diseases? (NTDs or otherwise) - Does the current understanding of trachoma in these populations affect the delivery of TCPs |
|  | Perceptions of trachoma and TCPs in marginalised communities/Maasai populations | - What are some of the healthcare beliefs on trachoma in the Maasai population or marginalised communities? - How accurate are the healthcare beliefs on trachoma with in the Maasai population? - Do these beliefs affect the implementation of TCPs? - Has there been anything done to change health beliefs by your organisation? - What are the biggest barriers in changing health beliefs in these populations? - What are some of the concerns of these communities have regarding trachoma? - Is there stigma around trachoma or TCPs in these communities? - Does stigma affect the implementation of TCPs? - How have these communities responded to TCPs? |
|  | Health Behaviours with trachoma | - What do the communities you work in do to deal with trachoma themselves? - Give an example? - Are these behaviours beneficial or not? - How well are these programmes being taken up in these communities? - Why are they being accepted (or not) in these communities? - Which changes in behaviours are accepted and which aren’t, and for what reason? |
| Decision-making factors on the approach of delivery of trachoma control programmes | Governance of the Organisation | - What aspects of trachoma-control does your organisation focuses on? - Why has your organisation chosen to focus on these aspects? - What aspects have worked well for your organisation, and why? - How effective do you feel your organisation is in dealing with trachoma? |
|  | Management of the Organisation | - How does leadership affect the delivery of TCPs? - What makes for an effective leader in the delivery of TCP? - What are some of the problems in leadership when delivering TCPs? - How are resources managed and distributed by your organisation? - Do you find the current method of management effective in dealing with trachoma? - Do you feel that the staff receives sufficient training to deal with trachoma effectively? - Do you feel that your organisation is receiving sufficient resources to deal with trachoma effectively? |
| The effect of different actors on the delivery of trachoma control programmes | Other NGOs | - How do you feel your organisation compares to others? - Are there any aspects of delivering TCP that has worked well for your NGOs compared to others? - Do you think that the delivery of your programmes in affected by other organisations? - Do you receive any outside support (excluding the Tanzanian Government)? |
|  | Government | - What is the role of the government in regards the delivery of TCPs? - Does the government influence the delivery of TCPs by your organisation? - Do you receive any government support? - Do you work with the government in planning TCPs? Examples? - Knowledge of other countries programmes? - Strengths and weakness of the programme here in Tanzania? - I understand in Tanzania that the National Trachoma control programmes/overseas… - How do you perceive working with the government? |
| General Improvement of the programmes | | - What are the main difficulties in implementing TCPs in these communities? - How do you think your organisation can improve the implementation of the TCPs whilst being mindful of the local culture and beliefs? - What would the organisation require in order to do so effectively?   - Research? Resources…? - What are the barriers to do so? |
